# Difficulty of predicting the presence of lymph node metastases in patients with clinical early stage gastric cancer: a case control study

**DOI:** 10.1186/s12885-015-1940-3

**Published:** 2015-12-01

**Authors:** Masatoshi Nakagawa, Yoon Young Choi, Ji Yeong An, Hyunsoo Chung, Sang Hyuk Seo, Hyun Beak Shin, Hui-Jae Bang, Shuangxi Li, Hyung-Il Kim, Jae-Ho Cheong, Woo Jin Hyung, Sung Hoon Noh

**Affiliations:** Department of Surgery, Yonsei University Health System, Yonsei University College of Medicine, 50 Yonsei-Ro, Seodaemun-gu, 120-752 Seoul, South Korea; Brain Korea 21 PLUS Project for Medical Science, Yonsei University Health System, Yonsei University College of Medicine, Seoul, Republic of Korea; Department of Internal Medicine, Institute of Gastroenterology, Yonsei University Health System, Yonsei University College of Medicine, Seoul, Republic of Korea; Department of Gastric Surgery, Tokyo Medical and Dental University, Tokyo, Japan; Department of Gastrointestinal Surgery, Peking University School of Oncology, Beijing Cancer Hospital and Institute, Beijing, China; Department of Surgery, Samsung Medical Center, Sungkyunkwan University School of Medicine, Seoul, 06351 South Korea; Department of Surgery, Inje University Busan Paik Hospital, Inje University College of Medicine, Busan, South Korea

**Keywords:** Early gastric cancer, Lymph node metastasis, Preoperative prediction

## Abstract

**Background:**

The relationship between pathological factors and lymph node metastasis of pathological stage early gastric cancer has been extensively investigated. By contrast, the relationship between preoperative factors and lymph node metastasis of clinical stage early gastric cancer has not been investigated. The present study was to investigate discrepancies between preoperative and postoperative values.

**Methods:**

From January 2011 to December 2013, 1042 patients with clinical stage early gastric cancer who underwent gastrectomy with lymphadenectomy were enrolled. Preoperative and postoperative values were collected for subsequent analysis. Receiver operating characteristics curves were computed using independent predictive factors.

**Results:**

Several discrepancies were observed between preoperative and postoperative values, including existence of ulcer, gross type, and histology (all McNemar *p*-values were <0.001). Multivariate analyses identified the following independent predictive factors for lymph node metastasis: postoperative values including age (*p* = 0.002), tumor size (*p* < 0.001), and tumor depth (*p* < 0.001); preoperative values including age (*p* = 0.017), existence of ulcer (*p* = 0.037), tumor size (*p* = 0.009), and prediction of the presence of lymph node metastasis in computed tomography scans (*p* = 0.002). These postoperative and preoperative independent predictive factors produced areas under the receiver operating characteristics curves of 0.824 and 0.660, respectively.

**Conclusions:**

Surgeons need to be aware of limitations in preoperative predictions of the presence of lymph node metastasis for clinical stage early gastric cancer.

**Electronic supplementary material:**

The online version of this article (doi:10.1186/s12885-015-1940-3) contains supplementary material, which is available to authorized users.

## Background

Gastric cancer is the fifth most common cancer in the world and the third most common cause of cancer-related mortality [1]. The incidence of early gastric cancer is increasing especially in Korea and Japan because of improvements in endoscopic diagnosis and the national screening systems [2–5]. In Korea and Japan, early gastric cancer has an excellent prognosis after surgical treatment, with 5-year survival rates of more than 90 % [5]. Lymph node metastasis in early gastric cancer patients has been reported to occur in approximately 10–15 % of cases, and it is one of the strongest prognostic factors for patients with early gastric cancer [1, 6–8].

The final result of the Dutch trial concluded that gastrectomy with D2 lymphadenectomy is a standard surgical procedure for patients with gastric cancer [9]. According to the Japanese guideline, which was established based on numerous pathological data, standard D2 lymphadenectomy is recommended for clinical stage early gastric cancer patients with lymph node metastasis, and more limited lymphadenectomy such as D1 or D1+ can be options for patients with clinical stage early gastric cancer without lymph node metastasis [10]. However, preoperative prediction of the presence of lymph node metastasis for clinical stage early gastric cancer patients is challenging, and preoperative diagnosis carries some degree of inaccuracy, so surgeons always need to consider the possibility of overstaging and understaging. Understaging leads to insufficient treatment, which may exhaust the chance of a cure, whereas overstaging leads to overtreatment, which may increase morbidity and mortality and affect the postoperative quality of life. Although the relationship between pathological factors and lymph node metastasis of pathological stage early gastric cancer has been extensively investigated, the relationship between preoperative factors and lymph node metastasis of clinical stage early gastric cancer has not been investigated [11–15]. The level of discrepancy between preoperative and postoperative diagnostic values also is not well understood.

The purpose of the present study is to investigate the discrepancies between preoperative and postoperative diagnostic values and the relationship between preoperative diagnostic values and lymph node metastasis of clinical stage early gastric cancer.

## Methods

### Patients and data collection

From January 2011 to December 2013, 1093 patients with clinical stage early gastric cancer underwent gastrectomy with lymphadenectomy at Yonsei University Severance Hospital, Seoul, Korea. Clinical stage early gastric cancer is defined as a lesion which is preoperatively diagnosed as confined to the mucosa or submucosa, irrespective of the regional lymph node metastasis. Diagnosis is primarily performed using esophagogastroduodenoscopy (EGD) and computed tomography (CT) scans [16]. Patients with the following factors were excluded: no EGD (*n* = 16) or CT scan report (*n* = 2) in our hospital, CT scans performed after endoscopic submucosal dissection (*n* = 15), incomplete pathological report (*n* = 9), remnant gastric cancer (*n* = 5), and multiple lesions (*n* = 4). A total of 1042 patients were enrolled into the present cohort. The following preoperative values of patients were collected: age, gender, body mass index, tumor size, existence of ulcer, gross type, histology, tumor location, and neutrophil-lymphocyte ratio. EGD and CT scans were performed for all patients. Tumor detectability and prediction of the presence of lymph node metastasis by CT scans were recorded, and endoscopic ultrasonography (EUS) was performed for some patients to examine tumor depth and the presence of perigastric lymph node metastasis. The following postoperative values of patients were collected: tumor size, existence of ulcer, gross type, histology, tumor location, pathological tumor depth, and lymph node metastasis. This study was conducted in accordance with the Declaration of Helsinki and was approved by the Institutional Review Board of Yonsei University Severance Hospital, which waived the need for written informed consent from the participants (4-2014-0971).

### Preoperative diagnostic methods

Preoperative diagnosis of clinical stage early gastric cancer was conducted through preoperative examinations such as EGD, EUS, and CT scans. Tumor size was measured using both EGD and EUS. If lesion size was measured using both EGD and EUS, the larger measurement was recorded as the representative tumor size [17, 18].

Preoperative CT scan was performed with a multi-detector row CT scanner (Sensation 16 or 64; Siemens Medical Solutions, Germany). Patients were instructed to fast for at least 4 h before the examination. Patients were prepared by injecting 10 mg butylscopolamine bromide and giving 2 packs of effervescent granules for gastric hypotonia and distention. Scanning was performed from the diaphragm to the symphysis pubis, with the patient in a supine position. A dose of 120–150 ml contrast medium was administered intravenously at a rate of 3–4 ml/s using a power injector, and the images of arterial and portal phases were obtained. CT scanning parameters were as follows: beam collimation, 0.75 mm × 16 or 0.6 mm × 64; kVp/effective mA, 120/160; and gantry rotation time, 0.5 s. Axial and coronal images were reconstructed at 3 mm interval with a slice thickness of 3 mm. If thickening or enhanced gastric mucosa was observed, it was regarded as a detectable tumor. Prediction of the presence of lymph node metastasis was established if the node met two or more of the following criteria: (1) ≥8 mm diameter in the short-axis, (2) round shape, (3) enhancement on contrast-enhanced CT scans, or (4) necrosis.

EUS was performed with radial scanning echoendoscopy at 5–12 MHz (GF-UE260; Olympus Optical Co. Ltd., Tokyo, Japan). The assessment of T-stage with EUS was based on the generally accepted 5-layer sonographic structure of the gastric wall. Early gastric cancer lesions were located within the first three layers, whereas advanced gastric cancer tumors invaded to the fourth and fifth layers. The assessment of N-stage with EUS was based on the existence of metastatic perigastric lymph nodes. Prediction of the presence of lymph node metastasis was established if the node met two or more of the following criteria: (1) ≥10 mm in the short-axis, (2) round shape, (3) hypoechoic pattern, or (4) smooth border [19].

### Statistical methods

Continuous values were analyzed with mean, standard deviation, and range. Correspondence between preoperative and postoperative values was analyzed using McNemar and Kappa values. Univariate analyses were performed using logistic regression analysis. Multivariate analyses were performed using multiple logistic regression models with the forward likelihood ratio method. Pearson’s coefficient correlation was performed to identify the correlation between two continuous values. Linear regression analysis was performed to compensate missing preoperative tumor size based on postoperative tumor size. A *p*-value less than 0.05 was regarded as significant for all analyses. Statistical analyses were performed using SPSS version 19.0 software (IBM SPSS, Chicago, IL). Receiver operating characteristics curves were obtained by the probability of finally selected multivariate logistic regression models and the presence of lymph node metastasis; the area under the curve, sensitivity, specificity, positive predictive value, and negative predictive value were calculated using R version 3.0.1 (http://www.R-project.org/) using the “pROC” and “Optimal Cutpoints” packages and the cutoff point was determined by the *Youden* method [20].

## Results

### Patient data

Baseline characteristics of all patients are shown in Table 1. Mean age was 58.0 years, 625 patients (60.0 %) were male, and 417 patients (40.0 %) were female. In preoperative CT scans, the tumors of 210 patients (20.2 %) were detectable, and 42 patients (4.0 %) were suspected to have lymph node metastasis. Pathological evidence indicated that 74 patients (7.1 %) had lymph node metastasis, and 81 patients (7.8 %) were diagnosed with advanced gastric cancer even though each lesion was considered preoperatively as early gastric cancer.

**Table 1:** Baseline characteristics of all patients

Variable	Preoperative (number, %)	Postoperative (number, %)
Age (mean ± SD, range) (years)	58.0 ± 11.7 (26–87)	
BMI (mean ± SD, range) (kg/m^2^)	23.6 ± 3.0 (15.1 − 35.4)	
Gender		
Male	625 (60.0)	
Female	417 (40.0)	
Tumor size (mean ± SD, range) (mm)		
Size using EGD (*n* = 631)	16.1 ± 8.0 (2–60)	−
Size using EUS (*n* = 479)	16.4 ± 5.9 (3–40)	−
Pathological size	−	21.1 ± 14.8 (1–165)
Ulcer		
Positive	66 (6.3)	245 (23.5)
Negative	976 (93.7)	797 (76.5)
Tumor location		
Upper third	146 (14.0)	127 (12.2)
Middle or lower third	896 (86.0)	915 (87.8)
Gross type		
0-Ia	29 (2.8)	18 (1.7)
0-IIa	278 (26.7)	34 (3.3)
0-IIb	224 (21.5)	184 (17.7)
0-IIc	428 (41.1)	667 (64.0)
0-III	83 (8.0)	62 (6.0)
AGC	0	77 (7.4)
Histology		
Papillary	1 (0.1)	1 (0.1)
Well differentiated	165 (15.8)	125 (12.0)
Moderately differentiated	260 (25.0)	264 (25.3)
Poorly differentiated	252 (24.2)	273 (26.2)
Signet ring cell	364 (34.9)	364 (34.9)
Mucinous	0	2 (0.2)
Carcinoma with lymphoid stroma	0	13 (1.2)
Tumor detectability in CT scan		
Detectable	210 (20.2)	−
Undetectable	832 (79.8)	−
Presence of LMN in CT scan		
Suspected	42 (4.0)	−
Unsuspected	1000 (96.0)	−
NLR	2.00 ± 1.32 (0.09 − 29.56)	−
Tumor depth		
Mucosa	−	588 (56.4)
Submucosa	−	373 (36.0)
Proper muscle	−	56 (5.4)
Subserosa	−	10 (1.0)
Serosa invasion	−	15 (1.4)
Lymph node classification		
pN0	−	968 (92.9)
pN1	−	42 (4.0)
pN2	−	23 (2.2)
pN3a	−	7 (0.7)
pN3b	−	2 (0.2)
Count of retrieved lymph nodes (mean ± SD, range)	−	32.6 ± 11.9 (5–74)

*SD* standard deviation, *BMI* body-mass index, *EGD* esophagogastroduodenoscopy, *EUS* endoscopic ultrasonography, *AGC* advanced gastric cancer, *LNM* lymph node metastasis, *CT* computed tomography, *NLR* neutrophil-lymphocyte ratio

**Fig. 1:**
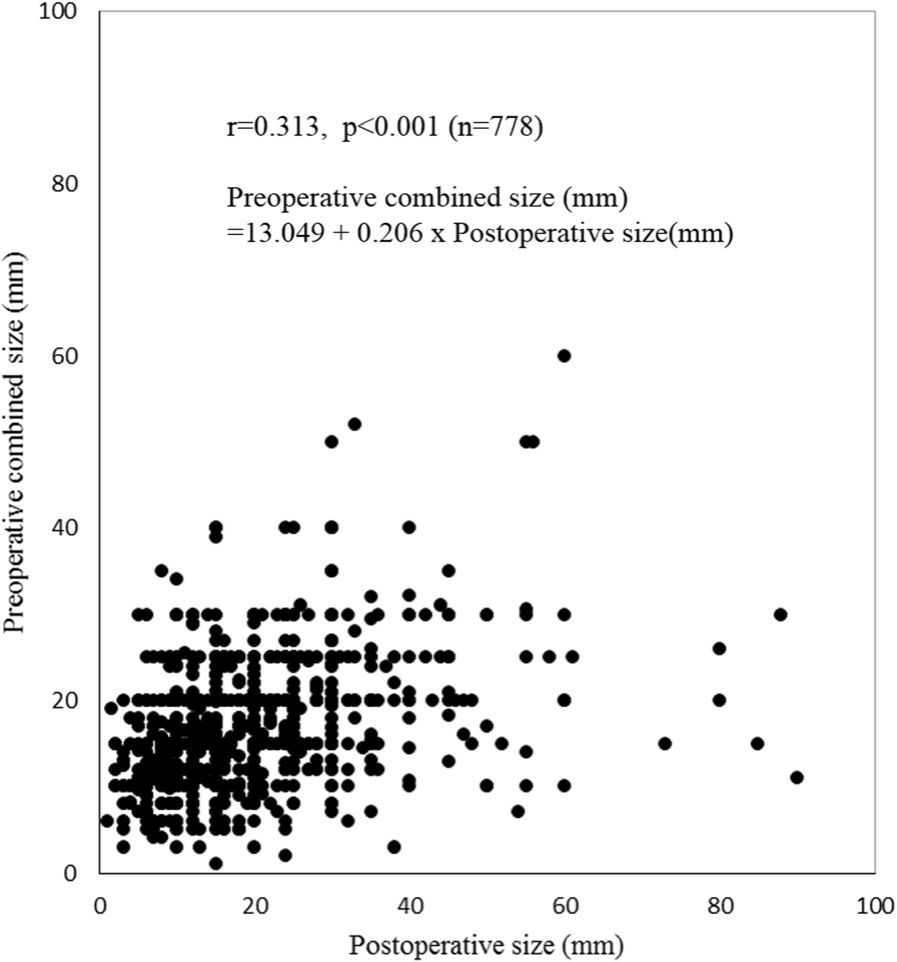
Scatter plot using postoperative and preoperative combined tumor sizes

Several discrepancies were observed between preoperative and postoperative diagnostic values including existence of ulcer, gross type, and histology (McNemar *p*-values were <0.001 for all results; κ and *p* for each diagnostic value were 0.082 and 0.001, 0.171 and <0.001, and 0.528 and <0.001, respectively; Table 2). The tumor size of each case was measured using EGD only in 299 cases (28.7 %), using EUS only in 147 cases (14.1 %), and using both EGD and EUS in 332 cases (31.9 %). Overall, the tumor size of 778 cases (74.7 %) was recorded as preoperative combined size. Correlation coefficients (*r* value) between pathological and preoperative tumor size using EGD (*n* = 631), EUS (*n* = 479), and combined size (*n* = 778) were 0.330, 0.264, and 0.325, respectively. Linear regression analysis of the relationship between pathological and preoperative combined tumor size also was performed [preoperative combined tumor size (mm) = 13.049 + 0.206 × Pathological tumor size (mm); *p* < 0.001, R^2=^0.110; Fig. 1). Missing data for preoperative combined tumor sizes [263 cases (25.2 %)] were compensated using the obtained formula (Additional file 1: Figure S1).

**Table 2:** Correspondence between preoperative and postoperative results regarding existence of ulcer, gross type, and histology

	Postoperative								*p*-value*
Preoperative	Ulcer		Negative			Positive			
	Negative		758			218			<0.001
	Positive		39			27			
	Gross type		I	IIa	IIb	IIc	III	AGC	
	I		10	3	4	6	1	5	<0.001
	IIa		4	16	47	175	15	21	
	IIb		1	5	79	121	4	14	
	IIc		2	9	46	324	21	26	
	III		1	2	8	41	21	11	
	AGC		0	0	0	0	0	0	
	Histology	Pap	WD	MD	PD	Muc	Sig	CLS	
	Pap	0	0	0	0	0	1	0	<0.001
	WD	0	92	62	8	0	3	0	
	MD	0	30	161	58	0	7	4	
	PD	0	3	34	141	1	65	8	
	Muc	0	0	0	0	0	0	0	
	Sig	1	0	7	66	1	288	1	
	CLS	0	0	0	0	0	0	0	

*p-values were obtained using McNemar analysis

*AGC* advanced gastric cancer, *Pap* papillary adenocarcinoma, *WD* well differentiated adenocarcinoma, *MD* moderately differentiated adenocarcinoma, *PD* poorly differentiated adenocarcinoma, *Muc* mucinous adenocarcinoma, *Sig* signet ring cell carcinoma, *CLS* carcinoma with lymphoid stroma

### Postoperative and preoperative predictive factors for lymph node metastasis

Univariate analysis for postoperative values indicated that age (*p* = 0.025), gross type (*p* < 0.001), histology (*p* = 0.001), tumor size (*p* < 0.001), and tumor depth (*p* < 0.001) were significant predictive factors for lymph node metastasis. Multivariate analysis indicated that age (*p* = 0.002), tumor size (*p* < 0.001), and tumor depth (*p* < 0.001) were independent predictive factors (Table 3).

**Table 3:** Univariate and multivariate analyses predicting LNM using postoperative values

Variable		Univariate		Multivariate	
		OR (95 % CI)	*p*-value	OR (95 % CI)	*p*-value
Gender	Male	1	0.118		
	Female	1.46 (0.91–2.34)			
Age^a^		0.98 (0.96–0.99)	0.025	0.97 (0.94–0.99)	0.002
BMI^a^		1.00 (0.93–1.08)	0.977		
Gross type	0-I, IIa	1	<0.001		
	IIb	0.51 (0.13–2.28)			
	IIc, III	0.92 (0.28–3.09)			
	AGC	8.33 (2.37–29.29)			
Histology	Pap, WD, MD	1	0.001		
	Muc, PD	2.65 (1.51–4.64)	0.001		
	Sig	0.86 (0.45–1.66)	0.654		
	CLS	0.0 (0.00–0.00)	0.999		
Ulcer	Negative	1	0.113		
	Positive	1.52 (0.91–2.54)			
Location	Upper third	1	0.145		
	Lower or middle third	0.63 (0.33–1.18)			
Tumor size^a^		1.03 (1.02–1.05)	<0.001	1.02 (1.01–1.04)	<0.001
T-stage	Mucosa	1	<0.001	1	<0.001
	Submucosa	5.13 (2.63–9.99)	<0.001	5.44 (2.76–10.71)	<0.001
	Proper muscle	11.73 (4.90–28.08)	<0.001	10.23 (4.21–24.84)	<0.001
	Subserosa	192.0 (36.82–1001.32)	<0.001	149.40 (27.21–820.33)	<0.001
	Serosa invasion	42.0 (13.11–134.56)	<0.001	28.62 (8.32–98.42)	<0.001

^a^Analyses were performed using continuous values

*LNM* lymph node metastasis, *OR* odds ratio, *CI* confidence interval, *BMI* body-mass index, *Pap* papillary adenocarcinoma, *WD* well differentiated adenocarcinoma, *MD* moderately differentiated adenocarcinoma, *Muc* mucinous adenocarcinoma, *PD* poorly differentiated adenocarcinoma, *Sig* signet cell ring carcinoma, *CLS* carcinoma with lymphoid stroma

Univariate analysis for preoperative values indicated that age (*p* = 0.025), existence of ulcer (*p* = 0.041), tumor size (*p* = 0.010), and prediction of the presence of lymph node metastasis in CT scans (*p* = 0.002) were significant predictive factors for lymph node metastasis. Multivariate analysis indicated that age (*p* = 0.017), existence of ulcer (*p* = 0.037), tumor size (*p* = 0.009), and prediction of the presence of lymph node metastasis in CT scans (*p* = 0.002) were independent predictive factors for lymph node metastasis (Table 4).

**Table 4:** Univariate and multivariate analyses predicting LNM using preoperative values

Variable		Univariate		Multivariate	
		OR (95 % CI)	*p*-value	OR (95 % CI)	*p*-value
Gender	Male	1	0.118		
	Female	1.46 (0.91–2.34)			
Age^a^		0.98 (0.96–0.99)	0.025	0.98 (0.96–0.99)	0.017
BMI^a^		1.00 (0.93–1.08)	0.977		
Gross type	0-I, IIa	1	0.585		
	IIb	0.70 (0.35–1.39)	0.304		
	IIc, III	0.86 (0.50–1.45)	0.563		
Histology	Pap, WD, MD	1	0.515		
	PD, Muc	1.36 (0.76–2.43)	0.300		
	Sig	1.00 (0.57–1.76)	0.991		
Ulcer	Negative	1	0.037	1	0.037
	Positive	2.21 (1.05–4.67)		2.25 (1.05–4.81)	
Location	Upper third	1	0.893		
	Lower or middle third	1.05 (0.54–2.09)			
Tumor size^a^		1.04 (1.01–1.07)	0.010	1.04 (1.01–1.07)	0.009
NLR^a^		1.08 (0.95–1.22)	0.228		
Tumor detectability in CT scan	Undetectable	1	0.355		
	Detectable	1.30 (0.75–2.26)			
Presence of LMN in CT scan	Unsuspected	1	0.001	1	0.002
	Suspected	3.92 (1.80–8.55)		3.57 (1.62–7.88)	

^a^Analyses were performed using continuous values

*LNM* lymph node metastasis, *OR* odds ratio, *CI* confidence interval, *BMI* body-mass index, *Pap* papillary adenocarcinoma, *WD* well differentiated adenocarcinoma, *MD* moderately differentiated adenocarcinoma, *Muc* mucinous adenocarcinoma, *PD* poorly differentiated adenocarcinoma, *Sig* signet cell ring carcinoma, *CLS* carcinoma with lymphoid stroma, *NLR* neutrophil-lymphocyte ratio, *CT* computed tomography

### Association between CT scans and EUS results and lymph node metastasis

The correspondence between preoperative CT scan and EUS results and pathological lymph node metastasis is shown in Table 5. CT scan results were obtained from all enrolled patients, whereas EUS results were available from 491 patients (47.1 %). Prediction of the presence of lymph node metastasis in CT scan was the only significant predictor for lymph node metastasis (*p* = 0.002). The sensitivity, specificity, positive predictive value, negative predictive value, overstaging, and understaging of preoperative prediction of the presence of lymph node metastasis by CT scan were 12.2, 96.6, 21.4, 93.5, 3.2, and 6.2 %, respectively (Additional file 2: Figure S2).

**Table 5:** Correspondence between preoperative CT and EUS results and pathological LNM

			LNM (−)	LNM (+)	*p*-value
CT	Tumor detectability	Undetectable	776 (80.2 %)	56 (75.7 %)	0.367
		Detectable	192 (19.8 %)	18 (24.3 %)	
	Presence of LNM	Unsuspected	935 (96.6 %)	65 (87.8 %)	0.002
		Suspected	33 (3.4 %)	9 (12.2 %)	
EUS^a^	Tumor depth	Mucosa	151 (33.0 %)	10 (30.3 %)	0.298
		Submucosa	283 (61.8 %)	20 (60.6 %)	
		Proper muscle	22 (4.8 %)	2 (6.1 %)	
		Serosa exposure	2 (0.4 %)	1 (3.0 %)	
	Presence of LNM	Unsuspected	442 (96.5 %)	30 (90.9 %)	0.128
		Suspected	16 (3.5 %)	3 (9.1 %)	

^a^Data from 491 patients were available

*LNM* lymph node metastasis, *CT* computed tomography, *EUS* endoscopic ultrasonography

### Receiver operating characteristics curves using independent predictive factors

**Fig. 2:**
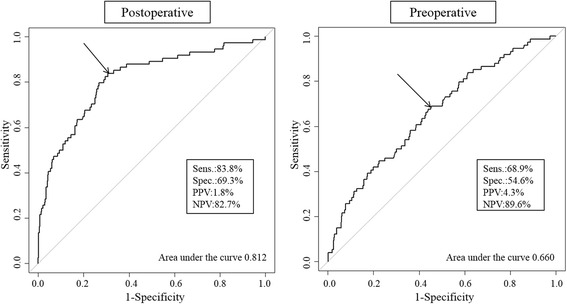
Receiver operating characteristics curves using postoperative (*left*) and preoperative (*right*) independent predictive factors for lymph node metastasis. Sens, sensitivity; Spec, specificity; PPV, positive predictive value; NPV, negative predictive value

Receiver operating characteristics curves were constructed by the probability of the finally selected logistic regression models in each postoperative (the model including age, tumor size, and T-stage, Table 3) and preoperative (the model including age, ulcer, tumor size, and prediction of the presence of lymph node metastasis in CT scan, Table 4) values and the event (lymph node metastasis). Figure 2 depicted the predictive performance of both multivariate models in postoperative and preoperative values.

Receiver operating characteristics were analyzed for area under the curve, sensitivity, specificity, positive predictive value, and negative predictive value using postoperative independent predictive factors, and were 0.824, 81.1 %, 71.4 %, 2.0 %, and 82.2 %, respectively. By contrast, area under the curve, sensitivity, specificity, positive predictive value, and negative predictive value of receiver operating characteristics using preoperative independent predictive factors were 0.660, 68.9 %, 54.6 %, 4.3 %, and 89.6 %, respectively.

## Discussion

We analyzed preoperative and postoperative predictive factors for the presence of lymph node metastasis in clinical stage early gastric cancer, and created prediction models using both independent preoperative and postoperative factors. The prediction model using postoperative independent factors was quite reliable (area under the curve = 0.812), whereas the one using preoperative factors was less reliable (area under the curve = 0.660). The prediction of the presence of lymph node metastasis in preoperative CT scan had the highest odds ratio among independent preoperative predictive factors; however, 3.2 % of patients were understaged and 6.2 % of patients were overstaged. Thus, CT scan is not reliable enough for prediction of the presence of lymph node metastasis, although it appears to be the most reliable tool in current practice.

One possible reason why preoperative values are not reliable enough to accurately predict the presence of lymph node metastasis is due to the discrepancy between preoperative and postoperative values. Postoperative tumor size is determined by measuring formalin-fixed specimens, whereas preoperative size is estimated from EGD or EUS results. The lesion border can be ambiguous, and it is challenging to accurately identify the lesion extent preoperatively. Tumor size measurement depends on the expertise of endoscopists. Postoperative specimens are fixed with formalin, which induces shrinkage of pathological samples. These factors often lead to discrepancies between preoperative and postoperative tumor size measurements [17, 18]. Histological heterogeneity is one of the distinctive characteristics of gastric cancer. There was usually a discrepancy between preoperative and postoperative histology results, which we also confirmed in the present study. The amount of tissue obtained through biopsy is usually limited, and it is taken primarily from mucosa, so the biopsy histology does not always represent the most dominant histology type of the lesion. According to the literature, the reported percentage of histological discrepancy in early gastric cancer ranges from 16.3 to 53.7 % [21–25]. This can explain why postoperative histology was significant for lymph node metastasis in the present study, whereas preoperative histology was not. Pathological T-stage is generally related to lymph node metastasis, which is why it is included in endoscopic submucosal dissection criteria [11]. If an accurate and precise preoperative assessment of tumor depth can be achieved, it would be helpful for predicting the presence of lymph node metastasis. However, this is still challenging, and preoperative diagnosis of tumor depth inherently contains some degree of inaccuracy even using EUS [26, 27].

Lymph node size is a common measurement when lymph nodes are assessed using CT scan. However, Monig et al. reported that mean diameter of metastatic lymph node was 6.0 mm, whereas that of tumor-free nodes was 4.1 mm. They also reported that the percentage of metastatic lymph nodes larger than 6 and 10 mm were 45 and 9.7 %, respectively, with a 10 % shrinkage factor during laboratory preparation [28]. A report by Park et al. on preoperative CT scans of pathologically lymph node metastatic-free patients concluded that lymph nodes larger than 8 mm in the short-axis can be detected in 14.9 % of early gastric cancer patients and 44.2 % of advanced gastric cancer patients. Those reports suggest that prediction of the presence of lymph node metastasis using CT scans cannot be completely accurate as long as criteria of the presence of lymph node metastasis include the lymph node size.

Alternative methods for prediction of the presence of lymph node metastasis include fluorodeoxyglucose positron emission tomography (FDG-PET) and EUS. FDG-PET is a preoperative diagnostic tool in various fields including gastric cancer. However, FDG-PET has low sensitivity, and is not currently a reliable tool for predicting the presence of lymph node metastasis and identifying early gastric cancer [29–31]. A meta-analysis by Cardoso et al. reported that the pooled accuracy of N-stage prediction by EUS was 64 % (95 % confidence interval = 43 − 84 %) [27]. EUS also was not a significantly reliable predictor in the current study. Therefore, FDG-PET and EUS cannot completely overcome the current lack of prediction accuracy.

Another method for prediction of the presence of lymph node metastasis is required. Sentinel node navigation surgery is a possible and promising solution. Application of sentinel node navigation surgery using dye-based or radioisotope-based techniques has been explored in the gastric cancer field [32–34]. Sentinel node navigation surgery using near-infrared imaging together with indocyanine green injection has been introduced to several fields including gastric cancer [35–39]. Optimized sentinel node navigation surgery should allow accurate detection of sentinel lymph nodes and real-time observation of lymphatic flow. However, a standard method for sentinel node navigation surgery has not yet been established, and the possibility of skip metastasis should always be considered. A multicenter randomized prospective clinical trial of sentinel node navigation surgery is ongoing in Korea to validate sentinel node navigation surgery for clinical application (NCT number 01804998) (https://clinicaltrials.gov/ct2/show/NCT018r04998?term=sentinel+and+gastric+cancer&rank=3). Therefore, a surgical strategy should be considered for each patient on a case-by-case basis according to current guidelines until accurate preoperative diagnostic methods for the presence of lymph node metastasis can be established.

The present study has several limitations. First, it is a retrospective study. Second, there was a selection bias because clinical stage early gastric cancer patients who underwent endoscopic submucosal dissection were not included unless their tumors met exclusion criteria for endoscopic submucosal dissection and required subsequent surgery. Third, precise information regarding preoperative tumor depth was not fully available because EUS was not performed for all patients in the cohort. Fourth, the preoperative tumor sizes of some patients were missing, although they were compensated using linear regression analysis.

## Conclusions

In conclusion, obvious discrepancies exist between preoperative and postoperative diagnostic values for the presence of lymph node metastasis for early gastric cancer. The prediction sensitivity and positive predictive value of the presence of lymph node metastasis using CT scan is low, but it currently remains as the most reliable tool. Predicting the presence of lymph node metastasis of clinical stage early gastric cancer is still challenging, and surgeons need to be aware of limitations in preoperative prediction accuracy of the presence of lymph node metastasis for early gastric cancer.

## Additional files

Additional file 1: Figure S1. Scheme showing how tumor sizes were obtained in the present study. EGC, early gastric cancer; EGD, esophagogastroduodenoscopy; CT, computed tomography; EUS, endoscopic ultrasonography. (TIF 73 kb) Additional file 2: Figure S2. Scheme showing the relationship between clinical and pathological lymph node assessments. cEGC: clinical stage early gastric cancer; cLN, clinical stage lymph node; pLN: pathological stage lymph node. (TIF 88 kb)

## Abbreviations

EGD: Esophagogastroduodenoscopy
CT: Computed tomography
EUS: Endoscopic ultrasonography
FDG-PET: Fluorodeoxyglucose positron emission tomography
