# Supplementary figures and images for: Difficulty of predicting the presence of lymph node metastases in patients with clinical early stage gastric cancer: a case control study
Source: BMC Cancer. 2015 Dec 1;15:943. doi: 10.1186/s12885-015-1940-3 (PMC4665830; doi:10.1186/s12885-015-1940-3)

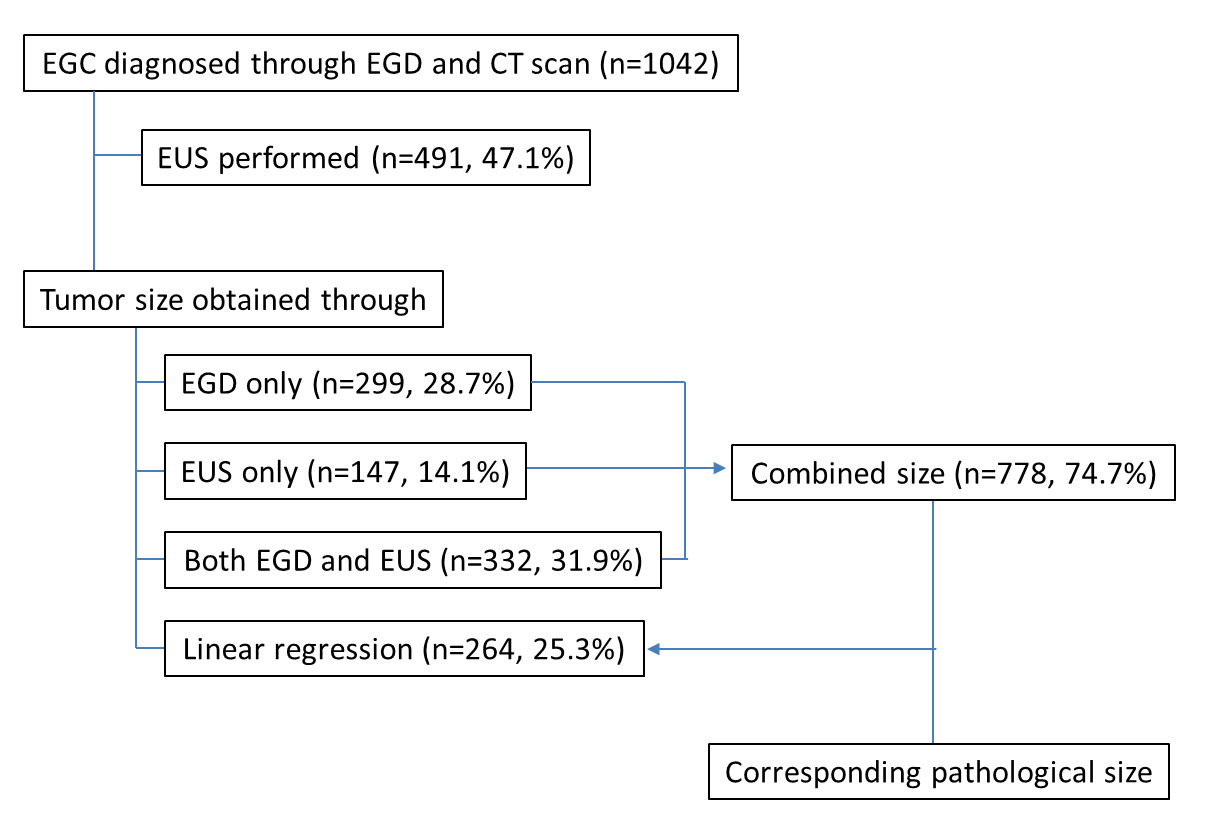

Supplement: Additional file 1: Figure S1. — Scheme showing how tumor sizes were obtained in the present study. EGC, early gastric cancer; EGD, esophagogastroduodenoscopy; CT, computed tomography; EUS, endoscopic ultrasonography. (TIF 73 kb) [file 12885_2015_1940_MOESM1_ESM.tif]

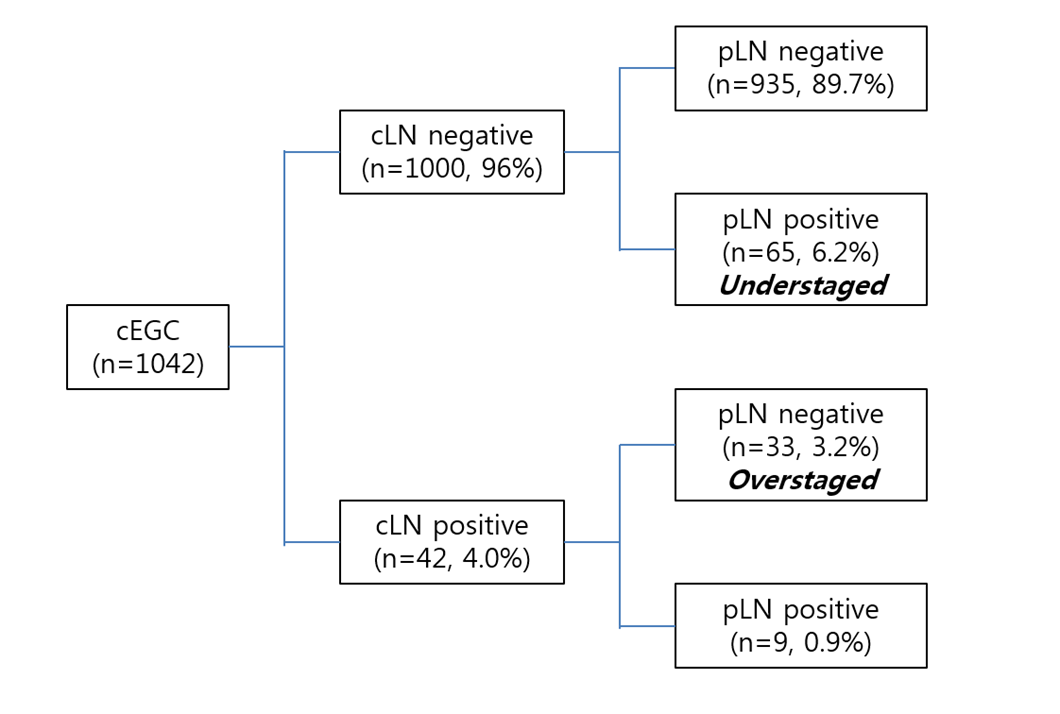

Supplement: Additional file 2: Figure S2. — Scheme showing the relationship between clinical and pathological lymph node assessments. cEGC: clinical stage early gastric cancer; cLN, clinical stage lymph node; pLN: pathological stage lymph node. (TIF 88 kb) [file 12885_2015_1940_MOESM2_ESM.tif]
